# Vitacrystallography: Appearance and Development of Cancer-Induced Structural Biomarkers in a Mouse Model

**DOI:** 10.3390/life15060904

**Published:** 2025-06-03

**Authors:** Oleksii Avdieiev, Sergey A. Denisov, Ashkan Ajeer, Lois Adams, Charlene Greenwood, Heather Nesbitt, Keith Thomas, Keith Rogers, Olga Solovyeva, Lev Mourokh, Pavel Lazarev

**Affiliations:** 1 EosDx UK Ltd., 5 New Street Square, London EC4A 3TW, UK; oavdieiev@matur.co.uk (O.A.);; 2 Institut de Chimie Physique, UMR8000, CNRS, Université Paris-Saclay, Bât. 349, 91405 Orsay, France; 3 School of Chemical and Physical Sciences, Keele University, Keele ST5 5BG, UK; a.ajeer@ucl.ac.uk (A.A.); c.e.greenwood@keele.ac.uk (C.G.); 4 School of Pharmacy and Pharmaceutical Sciences, Ulster University, Coleraine BT52 1SA, Co. Londonderry, Northern Ireland, UK; h.nesbitt@ulster.ac.uk; 5 School of Biomedical Sciences, Ulster University, Coleraine BT52 1SA, Co. Londonderry, Northern Ireland, UK; kg.thomas@ulster.ac.uk; 6 Shrivenham Campus, Cranfield University, Swindon SN6 8LA, UK; 7 Physics Department, Queens College, City University of New York, 65-30 Kissena Blvd, Flushing, NY 11367, USA

**Keywords:** vitacrystallography, X-ray scattering, structural biomarkers, mouse model, prostate cancer, cancer trajectory

## Abstract

Structural biomarkers determined by X-ray scattering of the tissues can complement conventional diagnostics and provide a pathway for early detection of diseases. In the present study, mouse models were utilized to observe the progression of prostate cancer. We induced cancer in the left lobe of the mouse prostate, whilst the right lobe was left uninoculated. The mice were sacrificed at increasing systematic time points, and lobe samples were subsequently analyzed using X-ray scattering. Control samples were also collected from healthy mice sacrificed at the same time points. This investigation revealed that the ratio between the X-ray scattering peaks associated with the lipids and water can serve as a structural biomarker of cancer, and this biomarker develops as the tumor advances. The obtained cancer trajectory can serve as a baseline for the determination of the disease stage, and the biomarker movement along the trajectory can be evidence of the healing or disease progression.

## 1. Introduction

Prostate cancer is the second most common cancer in men worldwide, with a lifetime risk of 1 in 9. It is primarily diagnosed in older men, with about 60% of cases in the U.S. occurring in those over age 65 [1]. At initial diagnosis, most cancer is confined to the prostate, although some cases progress to advanced stages, frequently spreading to organs such as the bones and liver. In 2022, there were almost 1.5 million new cases globally, and age-standardized incidence rates were similar in the US and UK at approximately 75 per 100,000. In the same year, there were 34,000 deaths in the U.S. and 13,000 in the UK as a result of prostate cancer [2]. Prostate cancer clearly presents a significant, continuing health and socioeconomic burden.

Generally, advances in our understanding of prostate cancer have required both in-depth comprehension of associated pathobiology and the development of new research platforms and models. Such models include those based on cell lines for genetic studies and drug screening. However, this *in vitro* work generally lacks a realistic representation of tumor heterogeneity and relevant tissue microenvironments. To address these limitations, *in vivo* murine models have been developed, providing a more complete depiction of prostate tumor microenvironments [3]. During the progression of prostate cancer through to metastatic stages, the tumor microenvironment undergoes significant modification, including changes in the extracellular matrix (ECM) and vasculature. Significantly, there appear to be similarities in stromal alterations between primary tumors and bone metastases [4], suggesting a critical role for the microenvironment (its architecture and components) in the effective disease progression. It has been well demonstrated that a particular challenge for prostate cancer therapy lies in its unique microenvironment, where, unusually, greater T-cell infiltration is correlated with poorer outcomes, as an example [5].

As with many complex pathologies, there are several gaps and challenges in understanding the etiology and progression of prostate cancer. Recently, new methods to study and characterize prostate tissues have been explored, enabling new understandings of metastatic change.

Several physical-science-based approaches (in contrast to genetics) have been developed for potential cancer diagnostic and monitoring probes. Of particular interest in the study of prostate cancers is mass spectrometry imaging, which has addressed issues of tissue heterogeneity and enabled the acquisition of lipid and metabolite profiles from different architectural prostate components, as well as their response to tumor progression [6]. Another method capable of mapping on relatively broad histological scales and increasingly used to address various diagnostic demands is Raman spectroscopy. This promising optical method with high spatial resolution for cancer diagnosis and treatment monitoring has matured to clinical application [7]. When applied to prostate cancer, it has indicated increased lipid synthesis and intracellular lipid heterogeneity within cancer cells [8].

Perhaps a less mature analysis approach involves the measurement of X-ray scatter. X-rays are scattered whenever they pass through materials, which is the basis of crystallography. The fundamental experimental requirement is to quantify the scatter features when tissues are illuminated with small-diameter X-ray beams. This diagnostic probe provides a detailed characterization of long- and short-range molecular order in terms of features such as molecular repeat distances and magnitude of disorder. It has previously been used to characterize ECM components of a small range of tissue types, especially those containing fibrous proteins (e.g., collagens), lipids, and ectopic calcifications [9]. We refer to *structural biomarkers* as measurable physical changes in the molecular structure or supramolecular organization of biological tissues associated with physiological or pathological conditions. In the present study, cancer-induced modifications of tissue structure serve as examples of such structural biomarkers. These components exhibit specific features at certain values of the transfer momentum, *q*, and the cancer-induced modifications of these features can serve as indicators of changes at the atomic, molecular, or supramolecular level, pertinent to early cancer detection. Compared to conventional imaging or molecular diagnostic techniques, X-ray scattering offers a non-destructive and highly sensitive means of detecting subtle molecular-level and organizational changes, providing unique insights into tissue structure that might not be accessible through other modalities. As with other groups’ reports, the work herein does not directly explore causal relationships between cancer and the diffraction signatures but rather adopts a pragmatic approach to examine relevant correlations. Building upon this framework, we refer to the application of X-ray scattering and diffraction techniques to study molecular-level structural changes in biological tissues, whether crystalline, semi-crystalline, or non-crystalline, as *Vitacrystallography*. This approach has been successfully applied in our previous studies to investigate atypical collagens associated with breast tumors and ECM invasion [10,11], lipid structures in liver cancer [12], and alterations to triglycerides in breast cancer [13,14,15,16,17].

There is an increasing interest in cancer-associated lipid metabolism as it has been suggested that these molecules have a role in supporting the rapid growth and survival of tumors. This relationship was reported for the first time almost 60 years ago [18,19] and revisited recently [20,21,22,23,24]. Cancer cells modify the structure of fatty acids and significantly alter lipid metabolism. Moreover, *de novo* lipids, which are synthesized in the tumor, can differ from those in circulation. These changes in lipid profile can be used to establish biomarkers. In particular, it was revealed [13,14,15,16,17] that in human breast tissues, the peak at approximately *q* = 14 nm^−1^, corresponding to inter-fatty-acid molecular distances, is prominent in healthy samples, but its height reduces in cancerous samples. Concurrently, at approximately *q* = 20 nm^−1^, the magnitude of another peak, associated with the oxygen-oxygen distance in the tetrahedral structure of water, increases in cancerous tissues.

For the first time, our study takes the initial steps toward exploiting X-ray scattering to examine tissue ECM components associated with prostate cancer and its progression. We have used a mouse model to initiate prostate cancer and have subsequently tracked temporal changes (*cancer trajectory*) in adipose characteristics as measured by X-ray scattering. The results have been compared to those related to healthy mice, tracked across the same temporal periods. We have demonstrated that the structural biomarkers in the mouse prostate tissues are the same as in the human breast tissues, with the magnitudes of the peaks associated with the lipids and water being modified as the tumor advances.

## 2. Materials and Methods

### 2.1. Mouse Inoculation

C57BL/6 male mice were used in this study, undergoing prostatic implantation procedures to establish the experimental model. The mice were sourced from accredited animal facilities and housed under standard laboratory conditions. They had *ad libitum* access to a standard rodent diet and water. The average mouse weight was 25.5g ± 3.66. Mice were randomly allocated into groups using the randomization function within Microsoft Excel.

For the surgical procedure, mice were anesthetized via inhalation using isoflurane in an oxygen carrier to ensure adequate sedation and minimize discomfort. A small incision was made in the lower abdomen to expose the prostate gland. A total of 5 × 10^5^ luciferase-expressing RM-1 cells were suspended in 25 µL of sterile phosphate-buffered saline (PBS) to ensure homogenous cell distribution. The cell suspension was carefully injected into the left anterior prostate lobe using a 29-gauge BD needle to avoid tissue damage and leakage of the suspension.

To minimize stress on the animals and promote recovery, absorbable sutures were used to close the muscle layer, and the skin was closed with surgical clips. The mice were subsequently monitored until full recovery from anesthesia and observed daily for signs of distress, infection, or other complications.

### 2.2. Sample Preparation

On Days 2, 4, 7, and 16 post-surgery, animals were injected intraperitoneally with 100 µL of D-luciferin in PBS (15 mg/mL) prior to imaging. The animals were anesthetized, and whole-body imaging was performed using an IVIS imaging system to confirm tumor establishment before tissue collection. Animals were sacrificed at specific time points, and the prostate was excised and imaged *ex vivo* for further analysis.

When the entire prostate was removed, a suture was placed through the left vesicle to highlight the correct injection side, ensuring accurate orientation during subsequent analyses. Two healthy control mice were sacrificed at each time point, and their prostates were removed to serve as controls for structural analysis.

The prostate samples were snap-frozen in liquid nitrogen immediately after dissection to preserve tissue integrity. Samples were then stored at −80 °C until further processing. For transport, the frozen samples were packed with dry ice and shipped to the experimental team for detailed structural analysis using wide-angle X-ray scattering (WAXS).

### 2.3. XRD Measurements

Wide-angle X-ray scattering (WAXS) data were collected on beamline I22 at the Diamond Light Source [25], the UK’s national synchrotron facility. This facility provides high-brilliance X-ray beams ideal for structural analysis at the nanoscale. The experiments were conducted using monochromatic X-rays of 12.4 keV, selected to optimize scattering contrast. The beam size was finely tuned to approximately 240 × 60 µm to ensure high spatial resolution and to facilitate precise mapping of structural heterogeneity within the samples.

Data acquisition was carried out using a Pilatus P3-2M detector. WAXS measurements were performed at a calibrated sample-to-detector distance of 170.21 mm, providing a *q*-range of 0.72 to 45 nm^−1^. This range enabled the detection of both large-scale structural features and fine molecular details, which are essential for comprehensive structural characterization.

Samples were prepared in individual sample cells measuring 5.2 mm in diameter and 2 mm in thickness, embedded within a custom-designed 9 × 9 aperture aluminum gel-solution sample grid. This grid was engineered to provide mechanical stability, uniform thermal conditions, and precise alignment during measurements. Scotch tape was used as window material to seal each cell, ensuring a uniform sample thickness and maintaining consistent sample hydration throughout the analysis.

For systematic data collection, each sample was scanned as a 5 × 5 grid of measurement points covering an area of interest. Based on preliminary optimization, a step size of 500 µm was selected to balance spatial resolution and data acquisition efficiency. At each grid point, 10 data frames were recorded to improve statistical reliability and reduce random noise, with an acquisition time per frame of 100 ms.

The experimental conditions, including beam energy, sample environment, and data acquisition parameters, were rigorously controlled and documented to ensure reproducibility. All raw data were processed using standard reduction pipelines, with detailed calibration steps and correction algorithms applied to account for detector geometry, background scattering, and beam fluctuations. This comprehensive methodology facilitates the reproducibility of our results and supports future comparative studies.

### 2.4. Image Processing

**Figure 1:**
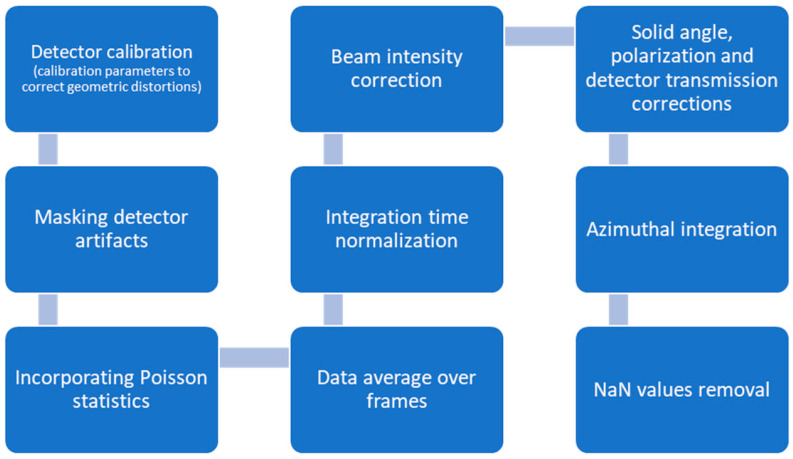
Schematic of image processing.

Image processing was conducted using the standard I22 pipelines [26] (systematic, pre-defined protocol steps for data processing) in conjunction with the DAWN data analysis software (Version 2.36.0) [27], designed to ensure the accuracy and reproducibility of the WAXS datasets. The processing workflow encompassed several key stages presented in Figure 1.

The process starts with the detector calibration. The calibration parameters are imported to correct geometric distortions inherent in the detector setup, ensuring accurate spatial mapping. Calibration was verified using standard reference materials to confirm detector alignment and pixel scaling precision. It is followed by mask generation, which involves applying an initial mask to exclude artifacts such as the beam stop shadow and non-responsive (dead) pixels. Consequently, the mask is dilated to eliminate edge effects and mitigate any residual problematic regions that could compromise data quality. Next is the error estimation, incorporating Poisson statistical models to represent photon counting statistics accurately. This process ensured robust uncertainty quantification, which is critical for the reliable interpretation of the diffraction data. After that, the beam stop diode readings are utilized for channel averaging to reduce random noise. Additionally, beam intensity correction and normalization by exposure time are applied to account for fluctuations in beam flux and measurement duration, enhancing data consistency. The next step is the frame processing, averaging the multiple frames collected at each measurement position to produce a single, high-fidelity 2D diffraction pattern. This stage includes background subtraction to isolate true sample-specific scattering signals from environmental or instrumental noise. It is followed by advanced corrections, applying intensity corrections for geometric distortions and sample absorption effects inherent to X-ray scatter measurements. Following these corrections, azimuthal integration is performed to convert 2D diffraction images into 1D intensity profiles, enabling detailed structural analysis. Finally, the non-numeric (NaN) values are removed, and the intensities are scaled, where necessary, to standardize the datasets. This step ensures that the processed data are suitable for subsequent quantitative analyses.

Following this rigorous processing pipeline, each measured position yielded a well-calibrated, background-corrected 1D diffraction profile suitable for quantitative structural analysis and comparative studies. The comprehensive documentation of each processing step enhances data reproducibility and facilitates the validation of analytical results across independent research efforts.

### 2.5. Data Processing

Following image processing, the dataset comprised azimuthally integrated 1D profiles obtained from 25 mice, distributed across five experimental groups: Control, Day 2, Day 4, Day 7, and Day 16, with five mice per group. Each profile contained 414 data points spanning *q*-ranges from 3.25 to 21 nm^−1^, representing measurements from both the left and right prostate sides. After meticulous manual data cleaning to remove artifacts and inconsistent readings, 564 individual measurements were retained for further analysis.

The 1D profiles underwent L1 normalization to standardize intensity scales across measurements, ensuring comparability while preserving the intrinsic relative intensity distribution within each profile. Preliminary insights into group-level structural variations were obtained by averaging profiles within each experimental group. These highlighted key differences and guided the identification of two prominent scattering peaks at approximately 13.5 and 18 nm^−1^ within the *q*-range of interest.

Subsequently, each of the 564 measurements underwent independent curve fitting using a composite model. This model incorporated two Gaussian functions as approximate profiles for the identified peaks and an amorphous scattering term characterized by an inverse *q* to the fourth power dependence:

$$y\left(q\right)=\sum_{i=1}^{3}A_{i}exp\left\{-{\left(q-\mu_{i}\right)}^{2}/2\sigma_{i}^{2}\right\}+B/q^{4}+C,$$

where $A_{i}$, $\mu_{i}$, and $\sigma_{i}$ denote the amplitude, position, and standard deviation of the Gaussian peaks, respectively. The parameter B quantifies the magnitude of the amorphous scattering, and C represents a constant background offset.

Fitting parameters derived from individual profiles were averaged within each experimental group to elucidate temporal trends in peak characteristics. This analysis revealed trajectories describing the evolution of structural features over time, corresponding to biological changes in the experimental conditions.

To ensure robust comparative analyses, control group measurements were temporally matched to corresponding experimental time points prior to averaging the fitting parameters. This matching strategy enabled precise tracking of parameter variations across different stages, facilitating the extraction of detailed structural information and providing critical insights into the temporal dynamics of the identified peaks.

## 3. Results

**Figure 2:**
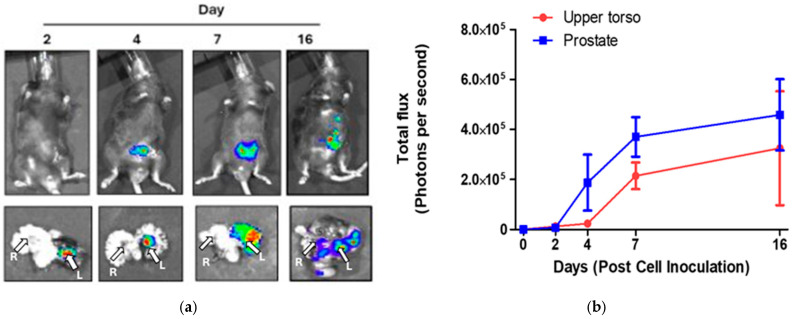
(**a**) Representative bioluminescence images of the whole body and excised prostate following RM-1 luciferase cells inoculation (5 × 10^5^) into the anterior prostate at Days 2, 4, 7, and 16. L and R indicate the left and right prostate lobes, respectively; (**b**) Bioluminescence flux measurements from the whole-body images, examining the ROIs of the upper torso and the prostate area of all mice.

To establish the cancer trajectory, prostate cancer was induced in mice through the inoculation of RM-1 luciferase-expressing cells into the left anterior prostate. Mice were sacrificed at specific time points—Days 2, 4, 7, and 16 post-inoculation—alongside control mice that did not receive cell inoculation. The luciferase expression allowed for the visualization of tumor development via D-luciferin injection prior to sacrifice. Representative bioluminescence images of the whole body and excised prostates are presented in Figure 2a, with the left prostate lobe positioned on the right of the pictures. The excised prostate visually showed an increase in tumor growth over the time period.

Bioluminescence flux measurements, quantified separately for the excised prostate and an upper torso region of interest (ROI) from the whole-body images, are depicted in Figure 2b. An increase in bioluminescence flux indicates progressive tumor growth, initially localized within the left lobe of the prostate before disseminating to other regions. Notably, the right prostate lobe remained largely unaffected, so it can be used as an internal control for subsequent analyses.

**Figure 3:**
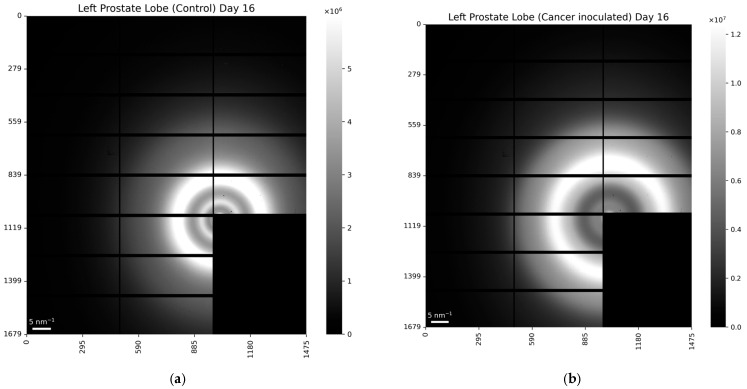
Typical WAXS images for (**a**) control and (**b**) cancer-induced mice. The scattering patterns show circularly symmetric intensity maxima with centers coincident with the primary X-ray beam (blocked by a beam-stop). The radius of each maximum is used to calculate the molecular repeat distances within the tissues.

Following dissection, the prostates of the inoculated and control mice were separated into the left and right lobes, with samples prepared for WAXS measurements on beamline I22 at Diamond Light Source [18]. Representative scattering patterns are displayed in Figure 3 for (a) the control and (b) inoculated mice. Azimuthal integration of these patterns yielded the intensity profiles as a function of the distance to the center, which, in turn, can be recalculated in terms of the momentum transfer as *q* = (4π sin *θ*)/*λ*, where 2*θ* is the scattering angle and tan 2*θ* is the ratio of the distances from the pixel to the center and from the sample to the detector.

**Figure 4:**
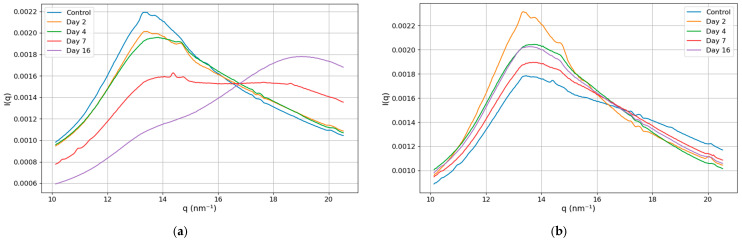
Intensity versus momentum transfer (*q* from 10 to 20 nm^−1^) for (**a**) the left and (**b**) the right prostate lobes at various post-inoculation time points.

Our analysis focused on a structural biomarker defined by the ratio of lipid and water peak intensities within the *q*-range of 10–20.5 nm^−1^. Figure 4 illustrates the intensity dependence on *q* for (a) the left and (b) the right prostate lobes across different time points. In the left lobe, notable changes in peak structure emerged as the tumor progressed. While the curves for Days 2 and 4 resembled those of the control group, by Day 7, the lipid and water peaks exhibited comparable magnitude. By Day 16, the water peak dominated, indicating advanced tumor progression. Conversely, the right lobe displayed minimal changes, with peak structures remaining consistent over time.

**Figure 5:**
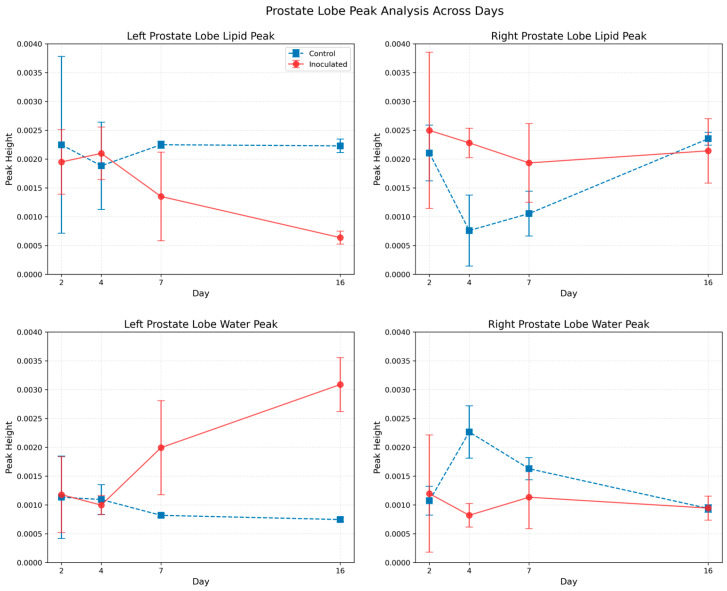
Peak magnitudes over time. Upper left panel: lipid peak for the left prostate lobe. Upper right panel: lipid peak for the right lobe. Lower left panel: water peak for the left lobe. Lower right panel: water peak for the right lobe.

To gain deeper insight, Gaussian curve fitting was performed on the lipid and water peaks, with results summarized in Figure 5, where the mean parts and standard deviations are shown for all groups containing five animals each. This analysis included data from control mice sacrificed at corresponding time points, facilitating robust comparisons. The fitting results confirmed that the lipid peak magnitude decreased in the tumor-affected left lobe, whereas the water peak magnitude increased as the tumor progressed. In contrast, both peaks remained relatively stable in the right lobe, underscoring its role as an internal control.

## 4. Discussion

Our findings demonstrate that prostate cancer progression in the mouse model can be effectively monitored through X-ray scattering patterns. The lipid-associated peak, corresponding to inter-fatty-acid molecular distances, exhibited a reduction in intensity as the tumor advanced. In contrast, the water-associated peak, reflecting the oxygen-oxygen distance, showed an increase in intensity. This dynamic interplay was quantified through a structural biomarker based on the ratio of the lipid and water peak intensities—an approach previously validated for the classification of human breast cancer samples [13,14,15,16,17]. Here, we extend its applicability to prostate cancer in a murine model, supporting the potential of this structural biomarker as a universal indicator of cancer, with evidence suggesting broad applicability across tissue types and species. Moreover, we tracked its modifications at various time points and established the corresponding cancer trajectory.

Several implications arise from our study. First, the conservation of structural biomarkers across species underscores their translational relevance. Although animal models provide experimental flexibility beyond ethical constraints applicable to human studies, the extrapolation of studies can be challenging. X-ray scattering addresses the molecular structure of tissue components (lipids, in our case), which is consistent between humans and animals. We showed that the response to cancer is the same as well, thereby reinforcing the validity of using animal models to explore human disease mechanisms.

Second, the universality of structural biomarkers across different organs was evident. Initially identified in human breast tissue, the biomarker reliably characterized prostate cancer in mice. The cross-organ consistency simplifies diagnostic workflows and supports the broader implementation of X-ray diffraction-based diagnostics. Notably, while our current data results were obtained in the synchrotron facilities, prior research [16,17] and our findings indicate that such biomarkers are detectable with laboratory-based X-ray diffractometers, expanding their accessibility for routine clinical applications. Recently, we showed that these structural biomarkers are visible for sample thicknesses up to 10 cm, making them applicable for in vivo measurements [28].

Finally, the established cancer trajectory presents a promising framework for evaluating treatment efficacy. Continuous monitoring of structural biomarkers could enable clinicians to track shifts toward or away from healthy baseline values, providing real-time feedback on therapeutic interventions. This approach may lead to more responsive, personalized treatment strategies, where decisions are informed by molecular-level changes rather than solely by morphological assessments.

Our current studies represent an initial step towards defining structural biomarkers, elucidating their temporal evolution (trajectory), and exploring their utility in cancer diagnosis and treatment. While our findings in the mouse model show promising conservation of structural biomarkers across species, translation into clinical diagnostics will require addressing challenges such as patient-specific tissue heterogeneity, variability in tumor microenvironments, and the adaptation of X-ray scattering techniques to clinical settings with appropriate low-dose instrumentation. Future research should focus on increasing sample sizes to improve statistical robustness, particularly addressing the variability observed in control samples. Additionally, expanding investigations to other organs within the mouse model will help assess biomarker universality further.

Exploration of additional biomarkers, including those related to triglycerides (at *q* = 1.5 nm^−1^), collagen, and glycoproteins, could enrich our understanding of cancer-related structural changes. Notably, our previous work in a canine model [29,30] demonstrated that the keratin structures in dog claws can serve as effective cancer biomarkers, highlighting the potential of X-ray scattering to uncover novel diagnostic indicators across species and tissue types. We believe that the structural biomarkers derived from X-ray scattering have the potential to drive transformative advances in cancer diagnostics, disease monitoring, and our broader understanding of biological structures and their functional implications.

## Data Availability

The files with the XRD patterns are available at https://doi.org/10.5281/zenodo.14751060, published 27 January 2025. The codes for the image processing are available upon request.
